# Bothersome tinnitus: Cognitive behavioral perspectives

**DOI:** 10.1007/s00106-018-0502-9

**Published:** 2018-04-30

**Authors:** R. F. F. Cima

**Affiliations:** Section Behavioural Medicine, Faculty of Psychology and Neuroscience, Maastricht University, 616, 6200 MD Maastricht, The Netherlands

**Keywords:** Hearing disorders, Auditory perception, Tinnitus, Psychological distress, Cognitive behavioral therapy, Hörstörungen, Auditorische Wahrnehmung, Tinnitus, Psychischer Distress, Kognitive Verhaltenstherapie

## Abstract

Tinnitus is *not traceable to a single* disease or pathology, but merely a symptom, which is distressing to some but not all individuals able to perceive it. The experience of tinnitus does not equate to tinnitus distress. Tinnitus suffering might be understood as a function of tinnitus-related distress in that* bothersome* tinnitus is an illness rather than a disease. In bothersome (distressing) tinnitus, the perception of the characteristic sound is a very disturbing and bothersome experience because of maladaptive psychological responses. Several cognitive and behavioral theoretical frameworks attempting to explain the nature and cause of tinnitus suffering have been introduced in and will be summarized here. Current treatment approaches are generally based on models that aim to: alleviate the perceptional experience by focusing on the tinnitus perception for habituation or even soothing purposes; decrease awareness of the sound by attentional training and cognitive interventions; decrease the maladaptive responses and the resulting distress by behavioral methods (i. e., exposure). The cognitive behavioral fear-avoidance model may offer an integrative cognitive behavioral approach that can lead to a new set of paradigms for studying the underlying mechanisms explaining chronic tinnitus suffering as well for developing innovative strategies to treat bothersome tinnitus.

## Zusammenfassung

Tinnitus ist nicht auf eine einzelne Krankheit oder pathologische Veränderung zurückzuführen, sondern nur ein Symptom, das für einige, aber nicht für alle Betroffenen belastend ist. Das Erleben von Tinnitus ist nicht gleichzusetzen mit tinnitusbedingtem Stress. Das Leiden am Tinnitus könnte insofern in Abhängigkeit von tinnitusbedingtem Stress verstanden werden, dass *störender* Tinnitus eher einen Zustand des Sich-krank-Fühlens als eine Krankheit darstellt. Bei störendem (stressverursachendem) Tinnitus wird die Wahrnehmung des typischen Geräusches – bedingt durch maladaptive psychische Reaktionen – als sehr störend und lästig empfunden. Verschiedene kognitive und verhaltensbezogene theoretische Konzepte, mit denen die Art und Ursache des Leidens am Tinnitus zu erklären versucht wird, sind etabliert und werden hier zusammengefasst. Aktuelle Therapieansätze basieren i. Allg. auf Modellen, deren Ziel es ist, die erlebte Wahrnehmung durch Fokussieren auf die Tinnituswahrnehmung zum Zweck der Habituierung oder gar Linderung zu erleichtern, das Bewusstsein für das Geräusch durch Aufmerksamkeitstraining und kognitive Interventionen zu senken oder die maladaptiven Reaktionen und den daraus resultierenden Stress durch verhaltensbezogene Methoden (d. h. Exposition) zu vermindern. Das kognitiv-behaviorale Angst-Vermeidungs-Modell („fear-avoidance model“) bietet möglicherweise einen integrativen kognitiv-behavioralen Ansatz, der zu neuen Paradigmen für die Untersuchung der zugrunde liegenden Mechanismen, durch die sich das Leiden am chronischen Tinnitus erklärt, sowie zur Entwicklung innovativer Strategien für die Behandlung des störenden Tinnitus führen kann.

The term “tinnitus” is usually defined as the continuous perception of sound in the absence of an external (or adequate) source. Although it describes the instance of perceiving a noise, this definition fails to recognize that for some individuals this perception coincides with severe anguish. Indeed, tinnitus is a fairly common “harmless” auditory sensation for up to 21% of the adult population [46]; however, an unfortunate subgroup (3–6%) [22, 26, 32] is afflicted by bothersome tinnitus, a signal predicting extreme distress and suffering [16].

Tinnitus is not a disease, but merely a symptom, as evidenced by the fact that the experience of tinnitus does not per se equate to tinnitus distress, which might add to the confusion regarding its terminology. Analogous to observations in chronic pain research [27, 56], one might view *bothersome* tinnitus as being an illness rather than a disease. A disease is defined as biological damage or malformation in tissue, anatomy, or physiology, whereas illness can be defined as the subjective experience of “being unwell” or “sick,” which concurs with the observation that tinnitus can cause, but is not by definition, a dysfunction that interferes with every aspect of daily living. In bothersome (distressing) tinnitus, the perception of the characteristic sound is a very disturbing and bothersome experience because of maladaptive psychological responses.

The symptom itself, *tinnitus aurium,* can be defined as the phantom perception of continuous sound or noise in the absence of an external (or adequate) source. In order to address and include the intrinsic psychological component of the distressing tinnitus experience, the following definition may be appropriate: A bothersome tinnitus (causing illness) might be best described as a negative auditory experience coinciding with aversive emotional reactivity, associated with, or described in terms of, actual or potential (bodily or psychological) harm. In analogy with the definition used for describing Chronic Pain suffering [50].

Confined to the individual’s subjective perceptual and emotional experience, tinnitus is not measurable or quantifiable by objective physical recordings, and is furthermore not traceable to disease, injury, or pathology in the brain or elsewhere. A medical or pharmacological cure is unavailable [23, 24] and audiometric perceptional properties of the tinnitus (the quality of the tinnitus sound, e. g., loudness or pitch) hardly predict the annoyance or severity of the tinnitus [2, 12, 36]. Perhaps counterintuitively, the more psychologically intrusive and threatening the sound becomes in the subjective experience of the individual, the more severe the suffering [12, 36]. Following these observations, tinnitus suffering might be best explained by psychological processes. There is evidence that cognitive misinterpretations, negative emotional reactivity, and dysfunctional attentional processes are of main importance in dysfunctional tinnitus habituation, leading to the severe tinnitus condition [4, 5, 7, 16, 25, 47, 59, 60].

Several cognitive and behavioral theoretical frameworks attempting to explain the nature and cause of tinnitus suffering have been introduced in the past and will be summarized here. Current treatment approaches are generally based on models that aim to: alleviate the perceptional experience by focusing on the tinnitus perception for habituation or even soothing purposes; decrease awareness of the sound by attentional training and cognitive interventions; or decrease the maladaptive responses and the resulting distress by behavioral methods (i. e., exposure). Current theoretical frameworks have been explanatory on some level, and the resulting treatment approaches have alleviated complaints leading to reports of occasional recovery to a satisfactory quality of daily life in many patients. The cognitive behavioral treatments (CBT) for tinnitus have indeed been shown to be effective in decreasing tinnitus distress, anxiety, and annoyance as well as improving daily life functioning [17, 35, 37, 39].

## Cognitive behavioral frameworks for tinnitus

### The habituation model

The habituation model proposed by Hallam and colleagues [28] is often considered the first attempt to offer a psychological account for troublesome tinnitus. It was proposed that the negative interpretation of the signal, and related heightened autonomic arousal levels, would lead to dysfunctional cognitive processing and therefore would disrupt habituation. Hallam purported that most people learn that the tinnitus sound is of low informational value and thus does not require a reaction. Consequently tinnitus does not pose a problem for the majority of people with living it. However, a bothersome and distressing tinnitus develops when these attentional processes malfunction, which is more likely at times of increased stress and arousal, which in turn restrains cognitive resources.

The model has remained largely theoretical, although tinnitus treatment approaches, such relaxation therapy, attention diversion techniques (directing attention away from tinnitus), and stress reduction by means of cognitive restructuring methods (aimed at altering beliefs about the tinnitus) have been based on its main premises [30]. Research to date has yielded mixed evidence in support of the habituation model [11].

### The neurophysiological model

The habituation model inspired Jastreboff [40, 43], who postulated that the association between tinnitus and an aversive emotional state emerges through classical conditioning. Classical (or Pavlovian) conditioning [51] refers to a process whereby two stimuli are presented together repeatedly (famously illustrated by the dog, presented with both a bell and meat). While doing this, an organism learns that the two stimuli are associated (i. e., “if bell, then meat”). Subsequent presentations of the principal stimulus alone (the bell, which is the conditioned stimulus), even without the meat (the unconditioned stimulus), proved to suffice to trigger the same response (salivating, which is the conditioned response).

**Fig. 1:**
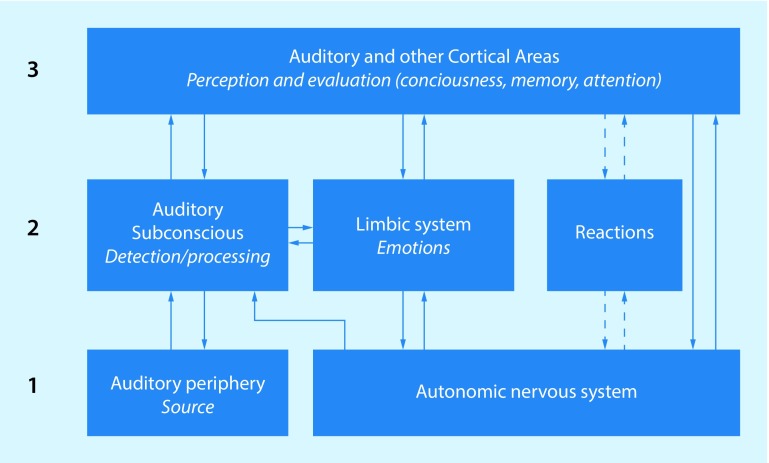
The neurophysiological model. (Adapted from Jastreboff [41])

The neurophysiological model (NP model) for chronic tinnitus is based on the premise that conditioned fear responses elicited by the tinnitus sound are the cause of the tinnitus becoming bothersome (Fig. 1; [40, 42]). This reasoning stems from animal research, in which conditioning paradigms were used to induce tinnitus-like fearful behavior in rats [43, 44]. The NP model distinguishes between three stages: (1) generation of the auditory stimulus in the auditory periphery; (2) detection of the tinnitus-related signal; (3) perception-evaluation of tinnitus. The NP model is mainly a model of tinnitus generation/detection, based on neurophysiological mechanisms.

### The cognitive behavioral model

**Fig. 2:**
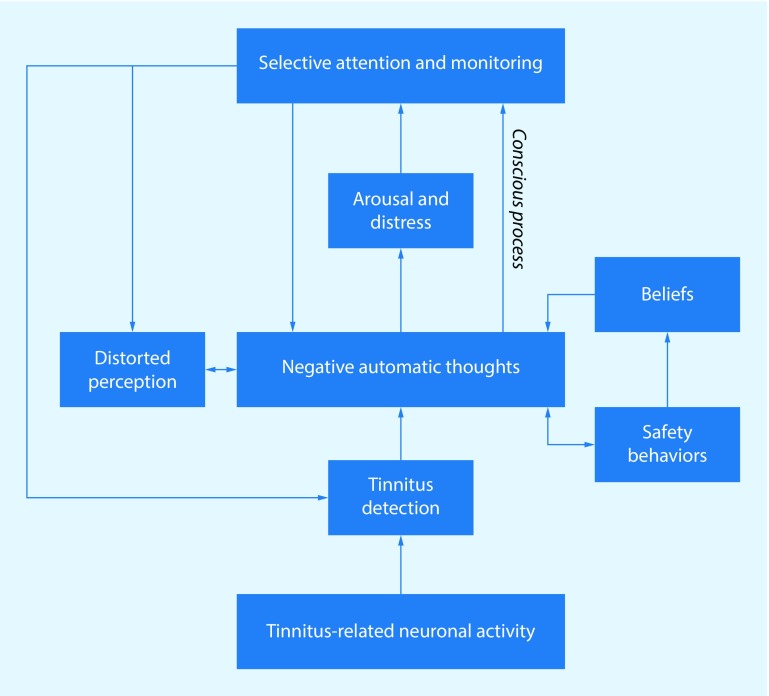
The cognitive behavioral model. (Adapted from McKenna et al. [49])

A recent conceptual model proposed by McKenna and colleagues [49] was based on a cognitive model of distress to explain insomnia [31]. McKenna et al. argue that mainly through negative cognitive misinterpretations of the tinnitus signal, distress and bodily arousal are provoked, leading to inaccurate evaluations of sensory activity and distorted perceptions (see Fig. 2). It is proposed that the resulting stress and hypervigilance contribute to a feedback cycle that exacerbates the distress associated with flawed sensory processing, of which tinnitus may be a major component. The model attributes a fundamental role to the negative evaluation of tinnitus. The negative evaluation of the tinnitus can be viewed as comprising primary and secondary appraisals.

Evidence exists that cognitive processes, such as interpretation, attention, and memory, are indeed involved in chronic tinnitus suffering [8, 19, 52, 54], although these studies were not specifically aimed at validating the model.

### The fear-avoidance model

**Fig. 3:**
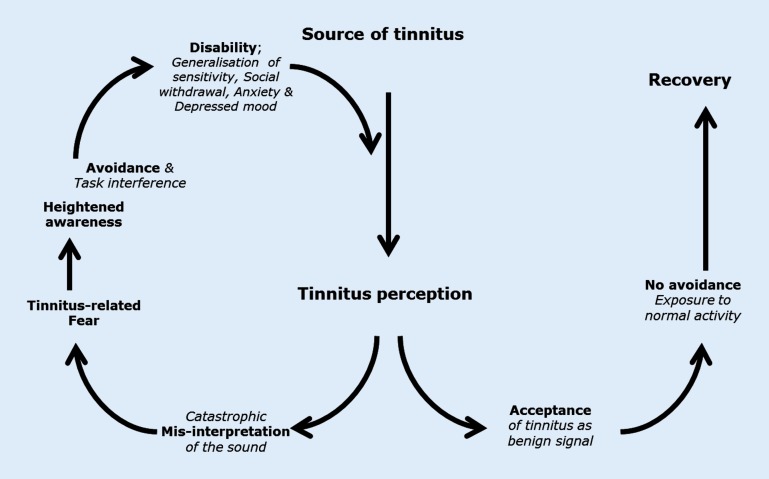
The fear-avoidance model of chronic tinnitus (based on the fear-avoidance model of chronic pain [57])

An alternative cognitive behavioral account for tinnitus has been postulated [15, 28, 45, 49], which is based on the fear-avoidance model (FA model) of chronic pain [57, 58]. The FA model (Fig. 3) for chronic tinnitus offers explanatory predictions about both the cognitive processes as well as the behavioral mechanisms. It predicts that individuals perceiving the tinnitus signal are subject to automatic emotional and sympathetic responses. These symptoms are misinterpreted as harmful or threatening. If the signal persists, the coinciding threatening (alarm) states, which indicate malignance of the signal, elicit conditioned—both classical and operant—fear responses, i. e., fear, increased attention, and safety seeking, i. e., avoidance and escape behaviors. These safety behaviors become negatively reinforced through instant decreased fear, which is adaptive in the acute phase. In other words, by avoiding or not exposing themselves to tinnitus-related perceptions, patients learn that their fear instantly diminishes. However, in the long run, through persistent avoidance of the tinnitus, tinnitus-eliciting, or tinnitus-increasing stimuli, the heightened fear and fear responses, such as hypervigilance and safety-seeking, are maintained. Avoidance behaviors subsequently lead to task interference and functional disability [13, 34]. A recent study supports the hypothesis that maintained high threat-expectancies and tinnitus-fear leads to increased tinnitus severity and distress, feeding into an endless circle of increased disability [18].

A typical feature of the FA model is that it predicts, next to the maladaptive pathway (leftward), an alternative, and more adaptive pathway (turning right), whereby a positive or neutral evaluation of the tinnitus results in no or low fear of the tinnitus, and no or lowered distress. In other words, the tinnitus sound is accepted by the system as being benign, therefore no unwanted attentional resources are needed, and in turn, avoidance and/or escape behaviors do not interfere with daily tasks; therefore, there is no severe disability due to tinnitus.

Accumulating evidence indicates that a CBT protocol for tinnitus based on this FA model, which targets re-appraisal of and exposure to the tinnitus sound, significantly reduces tinnitus distress, decreases tinnitus suffering, and improves the quality and daily functioning of tinnitus patients [1, 3, 6, 17, 35, 37, 48]. However, the cause–effect relationships of specific learning mechanisms are still unknown [15, 33, 45].

## Summary

The psychological impact of tinnitus has puzzled clinicians and scientists for many years. The extreme anguish and suffering of some patients are well observed and recorded, and the strong negative emotional connotation of the tinnitus experience seems to be commonly agreed upon [20, 21]. For this reason, cognitive behavioral theories and treatments have been applied in tinnitus research for decades [29, 53, 55] and CBT approaches for tinnitus have been repeatedly shown to be effective in decreasing tinnitus distress, anxiety, and annoyance as well as and improving daily life functioning.

Although there are common elements discernible across CBT-based treatments for tinnitus, CBT approaches vary largely, e. g., with respect to the content of treatment sessions (cognitive, behavioral, or both), number of treatment sessions, hours spent in therapy, group versus individual formats, face-to-face versus Internet based self-help therapies, combinations of different treatment elements, and tinnitus diagnostics and outcome assessments. Moreover, CBT treatments in general, and by extension in tinnitus practice and research as well, have evolved during the past 30 years, today often including elements of the so-called third-wave CBT interventions.

The theoretical frameworks have strong conceptual overlap and are based on the premise that the initially neutral tinnitus signal receives an “alarm” value, through classical conditioning. In turn, this negative tinnitus valence exacerbates negative responses in cognitions, emotions, and behaviors, hindering the normal process of habituation. Tinnitus distress ensues, which is the very negative and aversive state in which processes of adaptation and the efforts thereto have failed to return the organism to equilibrium or homeostasis.

It is important to note that treatment avenues sometimes seem to be contradictory. The NP model’s widely applied Tinnitus Retraining Therapy (TRT; [42]) approach suggests, next to extensive education, that a (partial) masking of the signal (avoidance of the signal, by avoiding silence at all costs) is the road to habituation; the habituation model and cognitive approaches purport that thought control and attention diversion techniques (alter thoughts/beliefs about the tinnitus and actively direct attention away from the tinnitus) will be beneficial for habituation. For short-term habituation, these strategies might work. On the other hand, the FA approach leads to an opposite treatment strategy, promoting exposure to tinnitus and eliminating avoidance tendencies (such as avoiding silence or directing attention away) in order to adjust threat expectancies and to decrease fear.

In the early years in CBT for tinnitus, a large part of treatment time was allocated to relaxation as a means of stress reduction as well as an attention-diversion method. In addition, emphasis was placed on cognitive processes. Control techniques and attention refocusing training, as well as the promotion of masking the tinnitus by sound enrichment (purported to increase control over tinnitus) were claimed to be essential in tinnitus control and therefore relief. These techniques were indeed helpful in the short term. Later on, other cognitive and behavioral components entered the CBT protocols for tinnitus, which promoted to decrease avoidance behaviors toward the tinnitus experience to decrease fearful reactions. The recent emphasis on countering avoidance behaviors, increasing moment-to-moment awareness, and being attentive toward tinnitus are illustrated in the growing application of exposure-based CBT elements and the use of third-wave CBT interventions, such as acceptance and commitment therapy (ACT) and mindfulness, in tinnitus health care.

Throughout the literature on effective tinnitus management, it is hard to find either CBT or sound-based approaches as the sole treatment strategy. In order to effectively manage complex tinnitus problems, treatments usually consist of a mixture of treatment approaches, combining psychologically informed education, sound therapy, and CBT approaches, have been proposed to effectively reduce the impact of the tinnitus on functioning [9, 10]. However, none of these have led to the implementation of one specific treatment strategy on a large scale, since research of sufficient methodological quality, generating comparable outcomes has been scarce [14, 38], thereby leaving patients and professionals alike with a myriad of options and combinations of treatment approaches.

The cognitive behavioral FA model might offer an integrative cognitive behavioral framework that can lead to a new set of paradigms for studying the underlying mechanisms of chronic tinnitus suffering. This FA approach integrates previous hypotheses and might prove helpful both for discovering new venues of investigation as well as offering a means of determining why not only cognitive but also behavioral treatment approaches are repeatedly found to be successful. The FA approach also offers a means for discerning which treatment-components work best for whom.

## Practical conclusion

- Tinnitus is not traceable to a single disease or pathology, but a symptom with a psychological impact that has puzzled clinicians and researchers for many years.
- Several cognitive and behavioral theoretical frameworks have been proposed to explain the nature, cause, and chronicity of tinnitus suffering.
- In the current report, the habituation model, the neurophysiological model, the cognitive behavioral model, and the fear-avoidance model are described, as well as the treatment methods arising from these.
- To effectively manage complex tinnitus problems, a combination of treatment approaches have been proposed to reduce the impact of tinnitus on daily functioning.
- The cognitive behavioral fear-avoidance model may offer an integrative approach that can help explain the underlying mechanisms of chronic tinnitus suffering and contribute toward the development of innovative strategies for treating bothersome tinnitus.
